# Magnetic-charge ordering and phase transitions in monopole-conserved square spin ice

**DOI:** 10.1038/srep15875

**Published:** 2015-10-29

**Authors:** Y.-L. Xie, Z.-Z. Du, Z.-B. Yan, J.-M. Liu

**Affiliations:** 1 Laboratory of Solid State Microstructures and Innovative Center of Advanced Microstructures, Nanjing University, Nanjing 210093, China; 2 Institute for Advanced Materials and Laboratory of Quantum Engineering and Materials, South China Normal University, Guangzhou 510006, China

## Abstract

Magnetic-charge ordering and corresponding magnetic/monopole phase transitions in spin ices are the emergent topics of condensed matter physics. In this work, we investigate a series of magnetic-charge (monopole) phase transitions in artificial square spin ice model using the conserved monopole density algorithm. It is revealed that the dynamics of low monopole density lattices is controlled by the effective Coulomb interaction and the Dirac string tension, leading to the monopole dimerization which is quite different from the dynamics of three-dimensional pyrochlore spin ice. The condensation of the monopole dimers into monopole crystals with staggered magnetic-charge order can be predicted clearly. For the high monopole density cases, the lattice undergoes two consecutive phase transitions from high-temperature paramagnetic/charge-disordered phase into staggered charge-ordered phase before eventually toward the long-range magnetically-ordered phase as the ground state which is of staggered charge order too. A phase diagram over the whole temperature-monopole density space, which exhibits a series of emergent spin and monopole ordered states, is presented.

Spin ices represent a class of highly interested frustrated magnetic systems in recent years and have been under intensive investigations for a series of fascinating ground states plus emergent thermodynamics and dynamics of fundamental excitations. The spin ice structures have been discovered in quite a number of pyrochlore oxide materials, such as Ho_2_Ti_2_O_7_^1^ and Dy_2_Ti_2_O_7_^2^. The large magnetic moments of rare earth ions (*μ* ~ 10 *μ*_*B*_ for Ho^3+^ and Dy^3+^) are sited on the corner-shared tetrahedrons and can be treated as Ising-like spins^3^, pointing along the local 〈111〉 axes due to the huge crystal field^4^. Spin ice is named for its unique ice states, in which two spins of every tetrahedron point into the tetrahedral center and the other two spins point out (i.e. so-called 2 in-2 out configuration). Such a spin alignment is reminiscent of proton positions in water ice following the so-called ‘ice rule’^5^, while a huge difference of spin ice from water ice is the remarkable fluctuations and flips of these spins, which generate a set of highly interested physical issues.

As one of these issues, it is found that due to the fluctuating nature of spins, the topological defects of the ice state with either 3 in-1 out or 1 in-3 out spin configurations are always present in realistic spin ices even at the sub-Kelvin temperature (*T*) range. Within the framework of dumbbell magnetostatic charge model^6^, the 2 in-2 out ice tetrahedrons are magnetic-charge neutral. These topological defects break the charge neutrality, enabling the lattice to carry magnetic charges of magnitude *Q* = 2 *μ*/*l*, where *l* is the distance between the centers of two nearest neighboring tetrahedrons. The states with such magnetic charges represent the elementary excitations in spin ice materials and behave similarly to the magnetic monopoles^6^. In consequence, the physics of magnetic monopoles has thus been receiving continuous attention since successful detection of them experimentally^7^.

Usually the density of monopoles in such spin ices is low and a single particle scenario or dilute monopole gas scenario may be sufficient. However, if two magnetic monopoles apart not very far from each other, their interaction becomes no longer negligible and has to be considered. Earlier work revealed that the inter-monopole interaction can be described by the Coulomb potential, and thus the monopole system can be viewed as magnetic Coulomb gas^6^. This concept allows an electrodynamic description of the monopole ground state and excitations in spin ice systems in a relatively simple and straightforward manner. For instance, it was claimed that a monopole system can be approximately treated as magnetic electrolyte^8^. Unfortunately, monopoles are dilute in realistic frozen ice states because formation of one monopole will cost vast amount of energy. Taking Dy_2_Ti_2_O_7_ as an example, one sees that the energy cost for creating one monopole-antimonopole pair is ~4.5 K, far higher than the temperature (~mK) at which a spin ice state can be reached. Therefore, so far only weakly correlated monopoles are considered in the theory of electrolyte models^9^,^10^, where the monopoles behave as ‘free’ particles in the diamond lattice. It was predicted that the motion of these ‘free’ monopoles in the lattice helps the evolution of the spin configuration to approach the true ground state^11^,^12^,^13^. Beyond this limit, approaches to obtain high monopole density in a spin ice lattice become emergent and highly appreciated.

In fact, quite a few of recent investigations have been devoted to possible roadmaps for controlling the monopole density, in particular in the low *T*-range^14^,^15^,^16^. In parallel, one of the core concerns is associated with the prediction that high density monopoles at low temperature can be strongly correlated and thus monopole condensation may be yielded^17^. The relevant symmetry of condensed monopole state and phase transitions become concerned issues. In fact, as the monopole fraction is up to ~30%, the spin lattice was predicted to develop the staggered monopole-ordered phase via a second-order transition^18^. As for enhancing the monopole density, it was recently suggested that high pressure induced lattice contraction is an effective roadmap, as experimentally demonstrated on Dy_2_Ge_2_O_7_^19^ as an incipient example. The underlying physics is related to the reduced chemical potential for monopoles and onset of monopole-antimonopole dimer pairs. It was revealed that a 1% lattice shrinkage, as observed in Ho_2_Ti_2_O_7_^20^, is sufficient to result in a 20% reduction in the monopole chemical potential^14^. Although this reduction is not yet enough to give rise to sufficient monopole density with which the monopoles are strongly correlated, this scheme is however promising since it is possible to increase the pressure so that more lattice shrinkage is obtained. Surely, even so, strongly correlated monopole phase in pyrochlore spin ice materials has not yet been observed experimentally.

An alternative and probably more efficient way to reach such strongly correlated monopole states, which seems challenging to realistic spin ice systems, may be found in artificial spin ice structures. In an artificial magnetic system, the ‘spins’ are artificially fabricated single magnetic nano-islands with huge magnetic moment, and they are aligned according to specific spatial symmetry^21^,^22^. Such a spin ice lattice is absolutely classical and the spin interaction can be easily modulated by the lattice symmetry and dimensionality^13^,^23^,^24^. More importantly, the monopoles and classical Dirac strings can be directly observed^25^, and careful treatment with the ‘spins’ allows us to observe the monopole excitations and dynamics in real space^24^,^26^,^27^.

Here we consider a common artificial spin ice lithographically created on a square structure, called square spin ice. It is a two-dimensional frustrated system mapped from a pyrochlore lattice by lattice projection onto the *ab* plane^28^. It is seen that a projection of the pyrochlore lattice into the square lattice reduces the 3-dimensional tetrahedral symmetry to the 2-dimensional square symmetry, while the coordination spin number for each vertex remains unchanged. The symmetry reduction gives rise to an energetic splitting of the six-fold degenerate ice states and allows a two-fold degenerate ground state^29^. This is the core of physics. By shifting properly the row and column ‘spins’ vertically, one can easily modulate the effective nearest neighbor interactions, and even obtain the single degeneracy degree in the ground state, as found in pyrochlore spin ices^13^,^24^,^28^. As a result, a full understanding of the 2-dimensional square spin ice will be very helpful for uncovering the nature of spin ice and provide an approach to study the collective phenomena. More specifically, the strongly correlated monopole states in pyrochlore spin ice, which remain highly concerned but far from clear, can be understood qualitatively in this simplified square spin ice lattice.

In fact, the thermodynamics and dynamics of monopoles in square spin ice lattice have already been investigated from several aspects. For example, by properly manipulating the magnetization of magnetic nano-islands, one can introduce monopoles and Dirac strings with variable density as topological defects in the lattice. By this way, the eigen-mode dynamics of these topological defects can be investigated^30^. On the other hand, theoretical studies predict that the density of thermally excited Dirac strings can be drastically increased by properly varying the vertical displacement of row spins and column spins^24^. This also enables an efficient manipulation of the monopole density in the lattice. However, so far no detailed work on the monopole correlation as a function of monopole density has been available. Even more, such a correlation induced phase transition and consequent monopole-ordered phase remain unexplored. Given the fact that the monopole density in artificial spin ices including the square spin ice lattice can be modulated by various practical approaches mentioned above, it is highly desirable to explore theoretically the monopole correlation and consequent phase transitions in a square spin ice lattice.

In this article, we intend to address the two issues by focusing on a square spin ice with anisotropic spin interaction. This anisotropic interaction will allow the square spin ice lattice the lowest degeneracy, while the lattice with isotropic spin interaction can be thus treated as a specific case. Such degeneracy-lift in three-dimensional pyrochlore spin ice which is isotropic can be realized by imposing a uniaxial pressure, as studied earlier^31^. We employ the Monte Carlo simulation to investigate square spin ice lattices with different monopole densities. Earlier simulation works already evidenced a few of simulation algorithms, including the loop spin flip algorithm^32^ and *Conserved monopoles algorithm* (CMA)^18^. The loop spin flip algorithm is efficient for overcoming local energy barriers without breaking the local topological structure. The CMA, introduced to address the issue of monopole interactions in spin ices, operates in a statistical ensemble with a fixed monopole density (*ρ*) in the lattice, so that the thermodynamic and dynamic aspects of monopole phase transitions can be characterized. We employ both algorithms in our simulations for various concerns.

The remaining part of this article is organized as follows. In Sec. 2, we introduce the square spin ice model with anisotropic spin interactions and the two algorithms for simulation. The main results are presented in Sec. 3, where the monopole gas and monopole crystals are treated separately. For the monopole fluid, the long-range Coulomb potential plays a central role in the dynamics of monopole accumulation and monopole correlation. A monopole dimerization effect in a certain temperature range will be demonstrated. For the monopole cluster, two phase transitions in the sequence towards the ground state will be revealed, and the intermediate monopole-ordered phase is charge-ordered state corresponding to that in three-dimensional pyrochlore spin ice. A brief conclusion will be given in Sec. 4.

## Model and Simulation Algorithms

### Hamiltonian of square spin ice lattice

**Figure 1:**
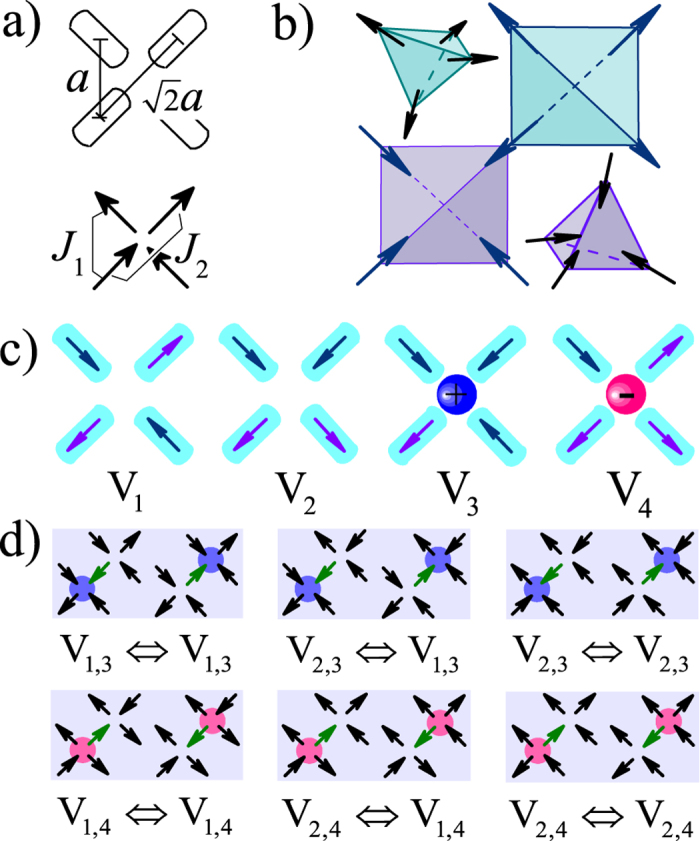
(**a**) The geometry of the square spin ice model. Each vertex consists of four spins. The distances between the vertical neighbors and parallel neighbors are *a* and √2*a* respectively, and the corresponding effective spin interactions are *J*_1_ and *J*_2_. (**b**) Two types of vertices: up-vertices and down-vertices. (**c**) Four different topological vertices in the monopole conserved model with V_1_ and V_2_ the vacuum vertices and V_3_ and V_4_ the monopole vertices with ‘+’ and ‘−’ charges respectively. (**d**) Possible six kinds of single spin flips obeying the rule of the conserved monopoles algorithm (CMA).

We consider a square spin ice in which single domain nano-islands are elaborately arranged on the gridlines of a two *L* × *L* dimensional square lattice^12^. Each nano-island has strongly anisotropic magnetic moment usually identified as Ising-like point spin for simplification. Each vertex of the lattice is surrounded by four spins, as shown in Fig. 1(a). Unlike the pyrochlore spin ice where the spin-spin separation in a tetrahedron is identical, the spin-spin separations along different directions in a vertex are different. Geometrically, for each vertex, the separation between two spins which are perpendicular to each other (abbreviated as vertical spin pairs hereafter) is *a*, and that between two spins which are parallel or antiparallel to each other (abbreviated as parallel spin pairs) is √2*a*, here *a* is the lattice constant. The spin at the *i*-th site is Ising-like with momentum 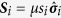, where unit vector 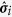 is the easy axis vector, *μ* is total moment, and *s*_*i*_ = ±1. For artificial spin ice, *μ* is a macroscopic quantity which is much bigger than the moment of a single ion.

It is noted that for pyrochlore spin ice, the vertices can be divided into two sets, as shown Fig. 1(b). The vertices with all the four spins pointing in are up-vertices (up-tetrahedra) and those with all the four spins pointing out are down-vertices (down-tetrahedra). For the square spin ice lattice here, each spin is surrounded by six nearest neighbors, of which four are the vertical spin pairs and two the parallel spin pairs. It is noted that for ideal point spins, the spin interaction takes the (***S***_*i*_·***S***_*j*_) form, and thus the vertical spin pair has zero exchange interaction. However, due to the fact that each spin in artificial spin ice is a nano-island which can’t be an ideal point spin, the geometrical shape of the nano-island will bring additional contribution to the exchange interaction between two artificial spins^13^. As an approach, an exchange interaction (*J*) will be imposed as an effective term to account of this contribution.

Furthermore, the long-range dipolar interaction is needed to take into account since it plays an important role for spin frustrated systems like spin ices. As a consequence, the magnetic Hamiltonian of the square spin ice model can be written as:


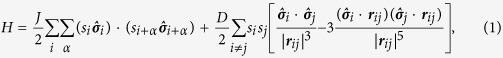


where sub-label *α* counts all the nearest-neighbor (vertical spin pairs) and diagonally next nearest neighbor (parallel spin pairs) exchange interactions, factor 1/2 here is introduced to avoid the repeated counting, and *J* is the exchange interaction coefficient. The second term is the long-range dipolar interaction, ***r***_*ij*_ is the distance between spins ***S***_*i*_ and ***S***_*j*_, ***r***_*ij*_ = ***r***_*j*_ − ***r***_*i*_, and *D* is the dipole-dipole coupling coefficient *D* = *μ*_0_*μ*^2^/(4*πa*^3^).

In realistic spin ices, the spin frustration comes from the nearest neighbor interactions. Thus, it is necessary to reduce equation (1) into the effective nearest neighbor Hamiltonian:


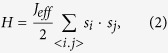


here *J*_eff_ is the effective exchange interaction and <*i*, *j*> implies the nearest-neighbor counting. Considering the two types of neighbors, *J*_eff_ takes two different values *J*_1_ and *J*_2_ (see Fig. 1(a)): *J*_1_ = 3/2*D* for all vertical spin pairs and 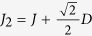 for all parallel spin pairs. Experimentally, the ratio *J*_2_/*J*_1_ can be easily changed by controlling the size and/or shape of nano-islands as well as offsets of the row spins and column spins in direction normal to the lattice plane^13^.

### Magnetic monopoles

Similar to the pyrochlore spin ice, the ice state of square spin ice lattice is fully occupied with the 2-in 2-out vertices over the whole lattice. This state is not unique in terms of the spin alignment with the vertex, as shown in Fig. 1(c), where the V_1_ vertex is non-magnetic with zero net magnetization, and the V_2_ vertex is ferromagnetic state with net magnetization along the upward or downward direction. The two types of vertices are called the vacuum vertices since they follow the ice rule. However, the two spin ice states, fully occupied respectively with the V_1_ vertices and V_2_ vertices, are non-degenerate and their energies per vertex unit are *E*_1_ = 2*J*_2_ − 4*J*_1_ and *E*_2 _= −2*J*_2_ respectively. The degeneracy can be achieved only at *J*_1_ = *J*_2_, i.e. *E*_1_ = *E*_2_, at which a fully spin frustration is reached. For the square spin ice lattice considered here, we usually take the case *J*_2 _< *J*_1_^13^,^33^,^34^ and then have *E*_1_ < *E*_2_, introducing an energy gap (Δ) between ice states V_1_ and V_2_, i.e. Δ = *E*_2_ − *E*_1_ = 4(*J*_1_ − *J*_2_) > 0. The positive Δ indicates the ice state with non-magnetic V_1_ state as the ground state, confirmed experimentally^29^.

In view of the dumbbell model^6^, both the V_1_ and V_2_ vertices are magnetic-charge neutral, implying that the ice states are vacuum states free of magnetic-charge. An excitation of the vacuum states may lead to the 3 in-1 out and/or 1 in-3 out vertex defects, breaking the magnetic-charge balance and creating magnetic monopoles with magnetic-charge *Q*_*α*_ = ±*q* on each vertex:


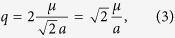


The two magnetic-charge vertices are shown in Fig. 1(c) too, where the V_3_ vertex has a positive charge denoted by blue solid spot (+) and the V_4_ vertex has a negative charge denoted by red solid dot (−). They are also called monopole and antimonopole respectively. It is clear that a square lattice can’t be fully occupied only with either the V_3_ vertex or V_4_ vertex. To keep the whole lattice charge-neutral, monopoles have to appear in pairs. Due to the long-range dipolar interaction, the monopole correlation (effective Coulomb interaction) can be described by the Coulomb’s law, i.e.:


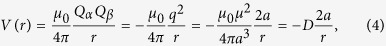


Here *r* is the separation between two monopole centers and an assumption *Q*_*α*_ = −*Q*_*β*_ is used for the last procedure.

### Simulation algorithm

We employ the Monte Carlo method to track the magnetic states and associated phase transitions in such dipolar square spin ice model with different monopole density *ρ*. In our simulations, we employ the CMA^18^ and more simulation details can be found in the Supplementary materials. This CMA imposes a set of constraint conditions. One is that there exist only four types of vertices in the simulation updating sequence, i.e. the V_1_ to V_4_ shown in Fig. 1(c). The V_1_ and V_2_ vertices obey the ice rule, but the V_3_ and V_4_ vertices are the excited states with a monopole defect of charge *q* and –*q* respectively. Obviously, one has *E*_1_ < *E*_2_ < 0 and *E*_3_ = *E*_4_ = 0 where *E*_3_ and *E*_4_ are the energy values for vertices V_3_ and V_4_. It is noted that the vertices with 4 in or 4 out are excluded due to the high formation energy. According to the CMA, any spin flip creating or destroying monopole is strictly forbidden and all the possible single spin flips are listed in Fig. 1(d).

## Results and Discussion

In our simulation, the monopole volume density *ρ* varies from *ρ* = 0 to *ρ* = 1, where the *ρ* = 0 and *ρ* = 1 states are two unique cases. Here *ρ* = 0 corresponds to the extreme case that there exists only one monopole pair (two monopoles with opposite charge signs) in the lattice. For the *ρ* = 1 extreme case, in order to allow the monopole movement, two vacuum vertices (i.e. V_1_ and V_2_ vertices) are allowed. For the spin ice model with conserved monopoles, the *ρ* = 0 and *ρ* = 1 cases are especially important, because the *ρ* = 0 case corresponds to the long-range V_1_ ground state for an infinite lattice, and we need to mention that the case *ρ* = 0 is a reference model to study the monopole interactions. For the *ρ* = 1 case, the monopoles are the most strongly correlated, similar to the dipolar kagome spin ice model^35^. Without losing the generality, here we present our simulated data at the energy gap Δ = 0.2*D* as an example, while varying the energy gap does not make the conclusion qualitatively different.

### Monopole dimerization

It is noted that experimental observation of the monopole excitations was realized in spin ice Dy_2_Ti_2_O_7_ at very low temperature (*T* ~ 0.1 K)^8^. At such a low *T*, the monopole density is certainly very close to zero, i.e. *ρ* ≪ 1. This is the first motivation for simulating the *ρ* ≪ 1 cases. It is also expected that at *ρ* ≪ 1, the vacuum state can’t be fully occupied only by the long-range ordered V_1_ vertices. The vacuum fluctuations (background) as induced by generation of low density V_2_ vertices become inevitable at a finite temperature. Such vacuum background must be accompanied with appearance of monopoles and antimonopoles. It will be shown that the lattice evolution with *ρ* ≪ 1 is significantly influenced by this vacuum background, probably leading to emergence of some new excited states such as monopole dimerization. This is the second motivation for simulating the *ρ* ≪ 1 cases.

We first investigate the *T*-dependent magnetic phase transitions. For *ρ* ≪ 1, the phase transitions depend mainly on the vacuum vertex configuration, noting again that the V_1_ vertex is non-magnetic, the V_2_ vertex is ferromagnetic, and the V_3_ and V_4_ vertices show also net magnetic moments. Since *E*_1_ < *E*_2_, a magnetic phase transition may occur upon decreasing *T* so that the low-*T* ordered state is filled with the V_1_ vertex. Here the magnetic order parameter *M* is defined by:


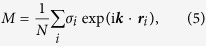


where ***k*** = (π/*a*, π/*a*) and *N* is the total number of spins, noting that *M* is not the magnetization. It is clear, for this ground state, we have *M* = 1.0.

**Figure 2:**
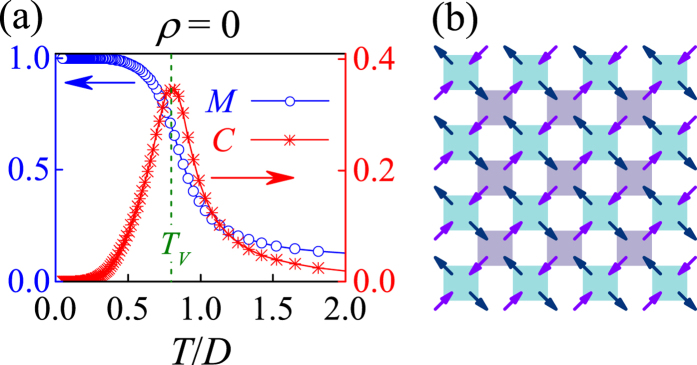
(**a**) Specific heat *C* and order parameter *M* as a function of *T* for *ρ* = 0 at *L* = 32. (**b**) A sketch of the vacuum ordered ground state.

**Figure 3:**
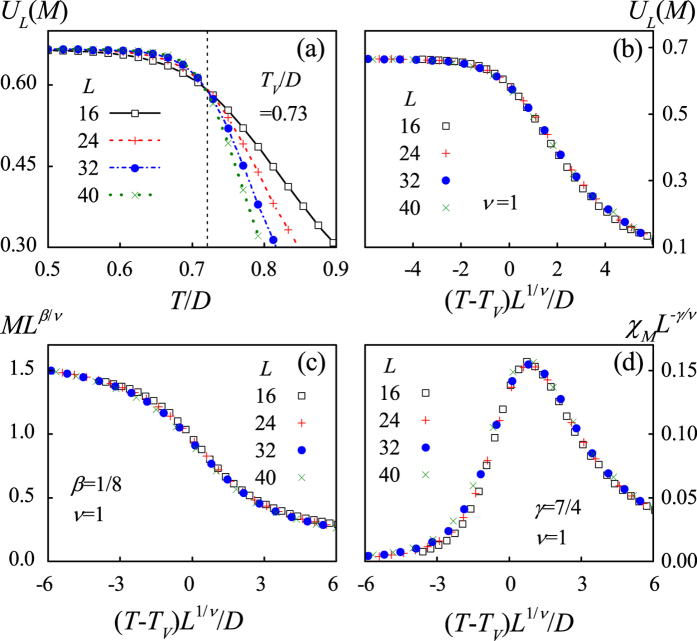
(**a**) The Binder’s fourth-order cumulant *U*_*L*_(*M*) = 1 − 〈*M*^4^〉/3〈*M*^2^〉^2^ for different *L* (=16, 24, 32, 40), giving the critical point *T*_*V*_ = 0.73*D*. The finite-size scaling analysis using parameters *U*_*L*_ (**b**), *M* (**c**), and *χ*_*M*_ = (〈*M*^2^〉 − 〈*M*〉^2^)/*Nk*_B_*T* (**c**). The critical exponents of the two-dimensional Ising model *β* = 1/8, *γ* = 7/4, *ν* = 1 are used here.

Since the vacuum vertices include only the V_1_ vertex and V_2_ vertex, it is expected that the phase transition from a disordered state to an ordered state is in the universality class of Ising model, as evidenced by the well-known universal behaviors of the transition by plotting the magnetic order parameter *M* and specific heat *C* as a function of *T* respectively for the case of *ρ* = 0, as shown in Fig. 2(a), with the phase transition point *T*_*V*_. The ground state lattice configuration is shown in Fig. 2(b). We also use the finite size scaling theory to check this phase transition, where the critical temperature point *T*_*M*_ is obtained from the intersection of the Binder’s fourth-order cumulants *U*_*L*_(*M*) (*U*_*L*_(*M*) = 1 − 〈*M*^4^〉/3〈*M*^2^〉^2^) of different lattice sizes, as shown in Fig. 3(a). The finite-size scaling on the *U*_*L*_(*M*), the *M*, and the susceptibility *χ*_*M*_(*T*) = (〈*M*^2^〉 − 〈*M*〉^2^)/*Nk*_B_*T* with *k*_B_ the Boltzmann constant, respectively, are plotted in Fig. 3(b)~(d), demonstrating the excellent data collapses upon the scaling using the critical exponents of the two-dimensional Ising model.

On the other hand, in this two-dimensional square spin ice system, the effective excitation energy of a monopole pair can be expressed as^36^:


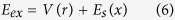


The first term is the usual Coulomb potential (equation (4)) and the second term is the excitation energy of the classical Dirac string. Furthermore we have


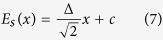


where *x* is the string length and *c* is the excitation energy of a nearest monopole-antimonopole pair. In our monopole conserved model, we have: (1) *c* = 0, (2) nonzero Dirac string tension given the nonzero energy gap Δ.

**Figure 4:**
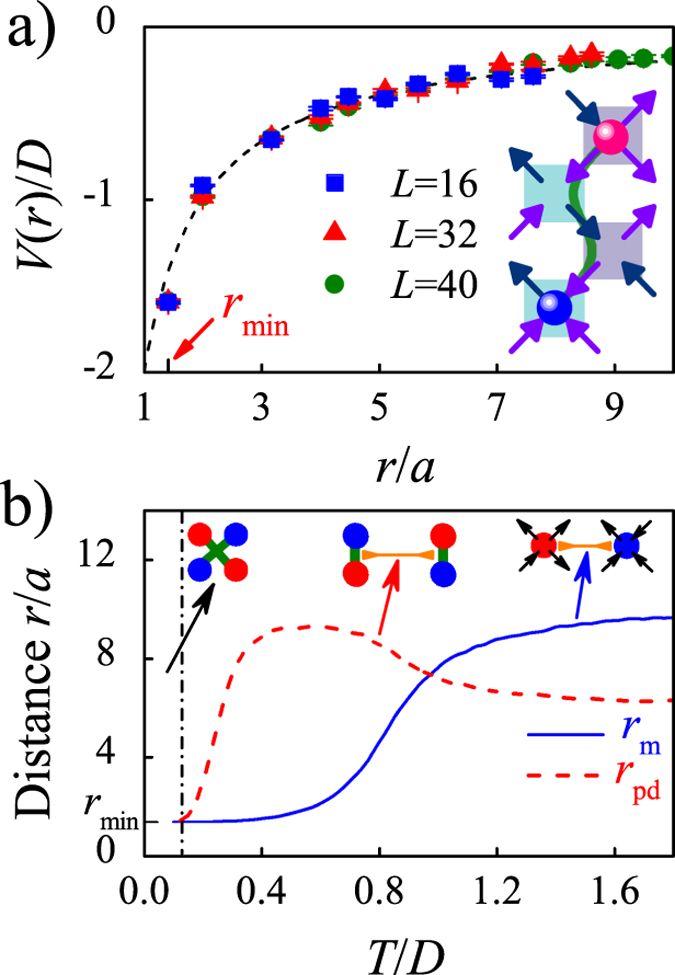
(**a**) The potential between two opposite monopoles, where the potential is simulated for *L* = 16, 32, and 40. The black dash line is the Coulomb potential fitted using the dumbbell model *V*(*r*) = −2*Da*/*r*. The insert is the sketch of two monopoles separated to a distance √10*a*. (**b**) The distance *r* between two monopoles (blue solid line) and between two monopole dimers (red dash line) as a function of *T* at *L* = 32.

For the cases *ρ* ≪ 1, the role of the Coulomb potential (equation (4)) and string energy (equation (7)) in controlling the monopole dynamics, if any, deserves for careful check. In conventional viewpoint, this role should be negligible since the monopoles in the lattice are very dilute. As a representative case, we consider the *ρ* = 0 lattice as an extreme case: two monopoles with opposite signs surrounded by the vacuum vertices. The Coulomb potential between the two monopoles can be obtained by calculating the lattice energy as a function of the separation (*r*) between the two monopoles, as shown in Fig. 4(a). In the simulation process, we first create a monopole pair (they are neighbored). According to the geometrical relationship shown in Fig. 1(a), the smallest distance between monopoles is *r*_min_ = √2*a*. Then we separate the two monopoles to a distance *r* via the single spin loop flip sequence that obeys the CMA’s rule each, followed by 10^4^ short loop flip events^32^ in order to reach a random vacuum environment around the monopoles. In this case, the two monopoles are fixed. Subsequently, the lattice energy (normalized by *L*^2^) is calculated. This process is repeated for 1000 times. The potential *V*(*r*) is obtained by averaging the 1000 independent sampling events. The evaluated *V*(*r*) curves for three lattices of different sizes (*L* = 16, 32, 40) are plotted in Fig. 4(a). It is seen that the calculated *V*(*r*) has a remarkably good agreement with the Coulomb potential (equation (4)), as expected from the dumbbell magnetostatic charge model. It should be stressed that the vacuum vertices are random chosen from V_1_ and V_2_ and the signal of the string energy (equation (7)) is lost in the background noise. Our results suggest that the magnetic monopoles interact via the effective Coulomb potential even in this square spin ice lattice, which is long-ranged and non-negligible even at *ρ* ≪ 1.

Obviously, in a cooling process, the monopoles with opposite signs in the lattice tend to attract due to the Coulomb attraction and the string tension. Once the distance between them reduces to the minimal, *r*_min_, they are strapped together, forming a neutral dimer. The Coulomb attraction between the new created monopole dimers should be much weak because of their charge neutrality and the string energy traps in the energy minima. In this circumstance, the properties of these neutral monopole dimers are much like gas molecules which can move randomly in the lattice. As a representative case, we consider a lattice where four monopoles (two monopole-antimonopole pairs) are assigned, where the two nearer opposite monopoles are regarded as a monopole-antimonopole pair with monopole-separation *r*_m_ ≥ *r*_*min*_. In order to understand the attraction between the two monopole-antimonopole pairs, we define their separation as *r*_pd_. In our simulations, parameters *r*_m_ and *r*_pd_ as a function of *T* respectively are calculated independently for an *L* = 32 lattice, as shown in Fig. 4(b), where the blue solid curve and red dashed curve represent the *r*_m_(*T*) and *r*_pd_(*T*) respectively. It is seen that the *r*_m_ falls down gradually with decreasing *T* in the high-*T* range, followed by a rapid fall within 0.6 < *T*/*D* < 1.2 before reaching down again slowly to the *r*_min_ at *T* < *T*_*V*_ (*T*_*V*_/*D* = 0.73 in our model). Obviously, this *r*_m_(*T*) dependence will suppress the attraction between the two monopole-antimonopole pairs (inter-pair attraction) at low *T*. Therefore, the *r*_pd_(*T*) exhibits distinctly different behavior from the *r*_m_(*T*), and there will appear a specific *T*-range (0.4 < *T*/*D* ~ 0.6 here) in which the *r*_pd_ reaches the maximal, due to the delicate competition between the inter-pair attraction and thermal fluctuations. In this *T*-range, the monopole-antimonopole pairs move more or less like the molecular Brownian motion. Surely, given further decreasing of *T* down to *T*/*D* < 0.4, the thermal fluctuations become insufficient against the inter-pair attraction, resulting in the rapid fall of the *r*_*pd*_ at *T*/*D* < 0.4. Eventually, a quadrupole or a tetramerization is reached due to the monopole condensation at *r*_pd_  → *r*_min_ at *T*/*D* ~ 0.1 and below.

**Figure 5:**
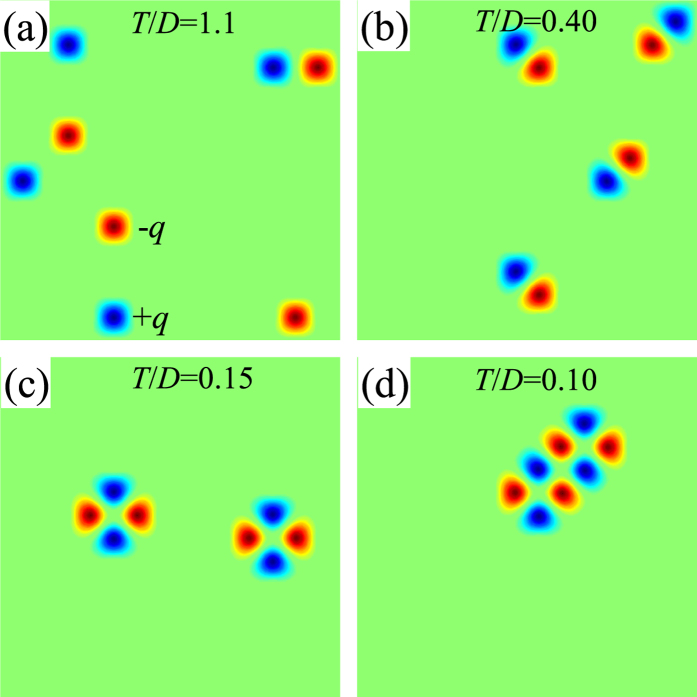
The contour plot of charge distribution on a 16 × 16 lattice: (**a**) monopole fluid, (**b**) monopole dimer, (**c**) monopole quadrupole metastable state, and (**d**) monopole crystal.

Such a dimerization sequence is spontaneous and the monopole condensation into monopole crystal becomes favorable if more monopole-antimonopole pairs are available. In a large scale, we show this dimerization process in Fig. 5 by the contour plot of the charge distribution in an *L* = 16 lattice where there exist four monopole pairs. The dimerization process with decreasing *T* is vividly illustrated. Actually, the four vertices around a single monopole vertex can’t satisfy the V_1_ arrangement simultaneously. However, for the dimer phase, the monopoles are bonded into dimer pairs, and the vertices around a dimer pair can satisfy the V_1_ arrangement simultaneously. The rotation and motion of a dimer pair can be realized by separating the two monopoles to a distance of 2*a* first and then getting them back to the *r*_min_. In Fig. 5(d), the dimer pairs are condensed into a small monopole crystal. Here, it is noted that in our simulation process there also exists a monopole quadrupole metastable state (Fig. 5(c)), whose energy is very close to the ground state.

### Monopole Crystal

Now we discuss another extreme case with monopole density *ρ* = 1. In this case, the lattice is fully filled with equal numbers of monopoles and antimonopoles, leaving only two vacuum vertices just for convenience of monopole movement. Surely, in this case the monopole interaction becomes significant since *E*_3_ = *E*_4_ = 0 and the nearest-neighbor interactions between spins are offset. In fact, a resemblance to our system is the dipolar kagome spin ice model^35^. In the kagome spin ice, each microstate obeying the ice rule always has one uncompensated charge, since every triangle vertex has odd number of spins (i.e. three spins). Every triangle must have either 2 in-1 out or 1 in-2 out arrangement, generating inevitably one uncompensated charge. As proposed by Chern *et al.*^37^, there exists an intermediate phase between the paramagnetic phase and the ordered magnetic phase in the dipolar kagome spin ice. Here we also observe two consecutive magnetic phase transitions in our model. Furthermore, an intuitive view suggests that the ground state contains two orders, i.e. the long-range magnetic order and staggered magnetic-charge order (SCO), noting that the long-range magnetic order accommodates spontaneously the staggered charge order, while the latter order (SCO) is not necessarily the consequence of the former order.

To characterize the SCO state, we introduce the staggered charge order parameter *Q*, which can be written as


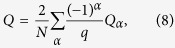


here *N* is the total spin number, *N*/2 is the number of vertices, and *α* is the indicator of the vertex type with the odd *α* representing the up-vertex and the even *α* the down-vertex. We then evaluate these two order parameters (*Q* and *M*) as a function of *T* for the case of *ρ* = 1 as an extreme case. It should be noted that the saturated magnetic order parameter *M* is 0.5 for the monopole crystal phase, which is smaller than that in the vacuum ordered phase having *M* = 1.

**Figure 6:**
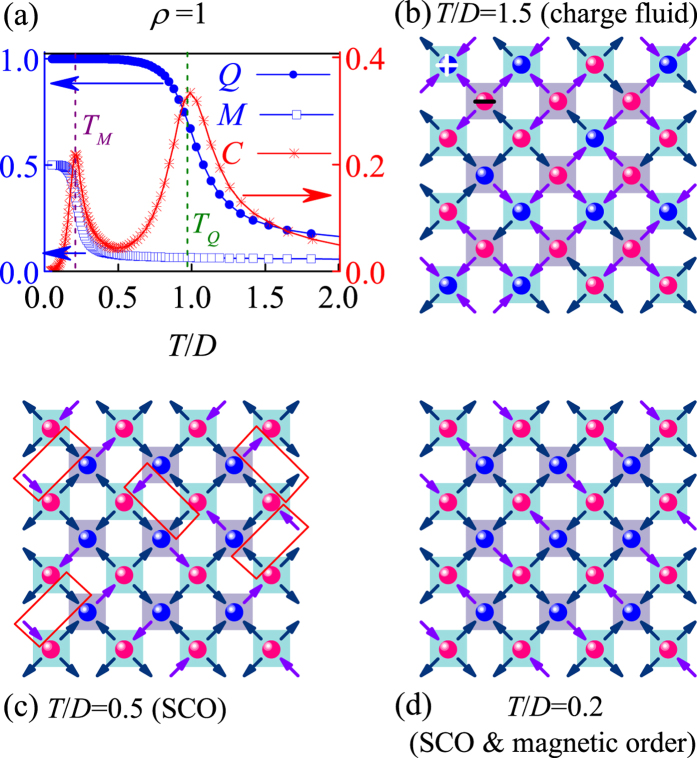
(**a**) Specific heat *C* and order parameters (*M* and *Q*) as a function of *T* for *ρ* = 1 at *L* = 32. Lattice configurations of the monopole fluid with no magnetic order and charge order (**b**) staggered charge order with no magnetic order (**c**) and ground state with long-range magnetic order and staggered charge order (**d**).

The calculated *C*(*T*), *M*(*T*), and *Q*(*T*) curves are plotted in Fig. 6(a) for *L* = 32. The two sharp peaks in the *C*(*T*) curve indicate the two consecutive phase transitions, with the higher phase transition corresponding to the staggered magnetic-charge ordering and the lower phase transition leading to the long-range magnetic ordering. In the high *T*-range, both the *M* and *Q* are close to zero, indicating a paramagnetic phase with neither magnetic order nor magnetic-charge order. A snapshot of the lattice is shown in Fig. 6(b) where the spins and charges are denoted by arrows and red/blue solid dots respectively. As *T* approaches *T*_*Q*_, the critical point for the magnetic-charge ordering, a rapid rise of parameter *Q* with decreasing *T* is observed. A representative lattice is shown in Fig. 6(c) where most of the spins along the two diagonal directions are antiparallel to each other but the rectangular boxes indicate the parallel spin alignment, i.e. the magnetic order is partial rather than long-ranged. A comparison of the spin configuration with that shown in Fig. 6(b) suggests the well-established staggered magnetic-charge order in spite of no long-range magnetic order. This partial magnetic order is favored at *T* < *T*_*Q*_ until the next critical temperature *T*_*M*_ at which the long-range magnetic ordering begins. As *T* < *T*_*M*_, both the *M* and *Q* rise up to the maximum (*M* = 0.5, *Q* = 1) and the corresponding lattice is shown in Fig. 6(d) at *T*/*D* = 0.2. The configuration in Fig. 6(a) is one feasible ground state and the whole lattice is occupied with the alternative stacking of the V_3_ and V_4_ vertices (red solid dots for negative charges and blue solid dots for positive charges).

**Figure 7:**
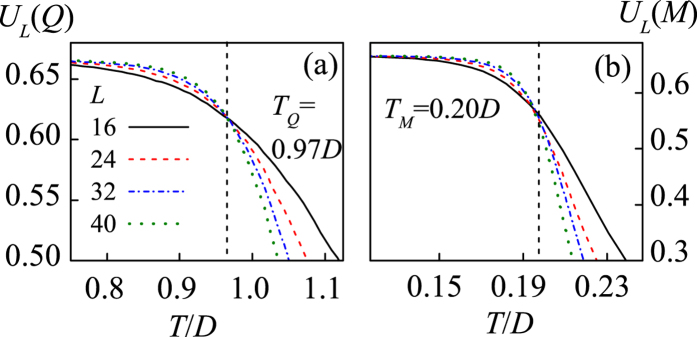
The Binder’s fourth-order cumulants *U*_*L*_(*M*) = 1 − 〈*M*^4^〉/3〈*M*^2^〉^2^ (**a**) and *U*_*L*_(*Q*) = 1 − 〈*Q*^4^〉/3〈*Q*^2^〉^2^ (**b**) at different *L*, giving the critical points *T*_*Q*_ = 0.97*D* and *T*_*M*_ = 0.20*D* respectively.

**Figure 8:**
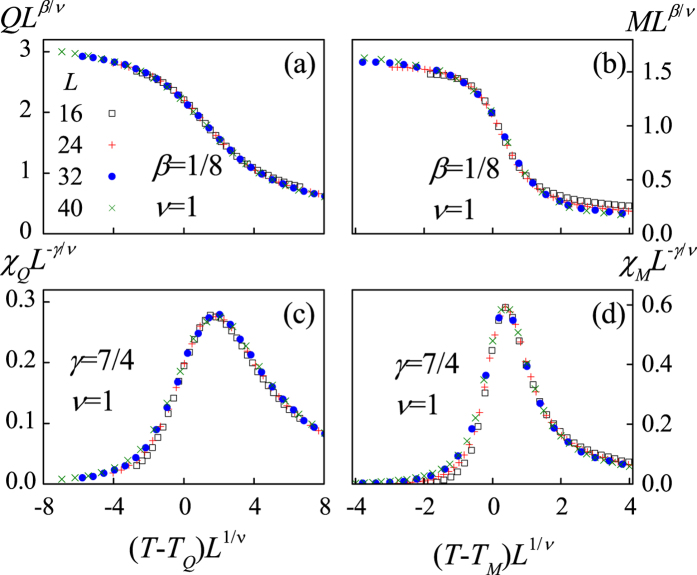
The finite-size scaling analysis using parameters *Q* (**a**) *χ*_*Q*_ = (〈*Q*^2^〉 − 〈*Q*〉^2^) /*Nk*_B_*T* (**b**) *M* (**c**) and *χ*_*M*_ = (〈*M*^2^〉 − 〈*M*〉^2^) /*Nk*_B_*T* (**d**) respectively. The critical exponents of the two-dimensional Ising model *β* = 1/8, *γ* = 7/4, *ν* = 1 are used.

The onset of such a partial magnetic-order in *T*_*M*_ < *T* < *T*_*Q*_ demonstrates that the monopole interactions are more prominent than the spin interactions, leading to *T*_*M*_ < *T*_*Q*_. To further characterize the two phase transitions, we consult to the Binder’s fourth-order cumulants *U*_*L*_(*Q*) = 1 − 〈*Q*^4^〉/3〈*Q*^2^〉^2^ and *U*_*L*_(*M*) = 1 − 〈*M*^4^〉/3〈*M*^2^〉^2^. The critical points *T*_*Q*_ and *T*_*M*_ are determined by checking the intersection of different *U*_*L*_(*Q*, *T*) and *U*_*L*_(*M*, *T*) lines, respectively, as shown in Fig. 7, giving rise to *T*_*Q*_/*D* = 0.97 and *T*_*M*_/*D* = 0.20. Similar to the case of *ρ* = 0, here each vertex is occupied by a monopole carrying either positive charge or negative charge, which should be in the 2-D Ising universality class too. As a result, the phase transition from the charge disorder to the charge order is expected to be Ising-like. The corresponding finite size scaling behaviors are presented in Fig. 8. Here we use the critical exponents of the Ising transition to rescale the *Q*(*T*) curves and charge susceptibility *χ*_*Q*_(*T*) curves for different *L*, where *χ*_*Q*_(*T*) = (〈*Q*^2^〉 − 〈*Q*〉^2^)/*Nk*_B_*T*. The excellent data collapse onto one master curve is demonstrated, as shown in Fig. 8(a,c).

In proceeding, it is noted that each charge-ordered state corresponds to four degenerate magnetic ground states, so the transition from the charge-ordered state to the long-range magnetic-order state is expected to be the same as that of the four-state clock model. Meanwhile, the phase transition of the four-state clock model is the Ising like^38^, allowing us to perform the finite-size scaling analysis on the transition from the charge-order to magnetic order. The results demonstrate the argument, as shown in Fig. 8(b,d) where all the rescaled data fall on the same master curve for both *M* and *χ*_*M*_.

### Phase diagram of the monopole conserved model

**Figure 9:**
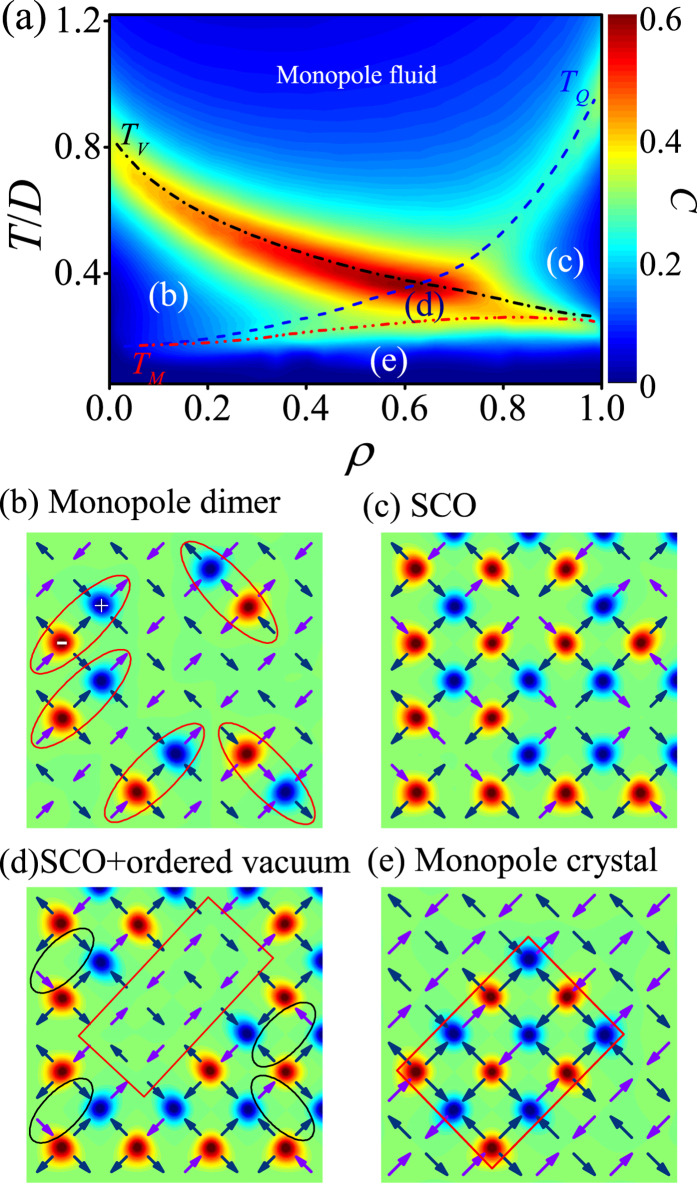
(**a**) Phase diagram of the monopole conserved model in the plane *ρ* vs *T*. The contrast scales the contour of specific heat *C*(*ρ*, *T*) at *L* = 32 for guide of eyes. The typical lattices for monopole dimer phase (**b**) staggered charge ordered (SCO) phase (**c**) SCO plus ordered vacuum phase (**d**) and monopole crystal phase (**e**) respectively.

Based on a comprehensive understanding of the two extreme cases and the monopole dimerization process, we are now in a good position to cover the whole region from *ρ*  ≫1 to *ρ* = 1. Extensive simulations on the whole region and associated phase transitions have been performed, giving rise to the *T-ρ* phase diagram shown in Fig. 9(a), where the color contrast reflects the magnitude of specific heat *C*(*ρ*, *T*), as indicated by the color bar aside and the curves *T*_V_ − *ρ*, *T*_Q_ − *ρ* and *T*_M_ − *ρ* are plotted for guide of eyes. The charge-ordered critical temperature *T*_*Q*_(*ρ*) increases with increasing *ρ*, indicating that the monopole correlation becomes stronger as the monopole density increases^17^. While the vacuum ordered critical temperature *T*_*V*_(*ρ*) exhibits distinctly different behavior from the *T*_*Q*_(*ρ*), the increasing monopoles weaken the spin interactions and result in the decrease of *T*_*V*_. In the phase diagram, the curve *T*_*V*_(*ρ*) intersects with *T*_*Q*_(*ρ*) at a critical monopole density *ρ*_*C*_ = 0.62. This phase diagram is divided into five sub-regions separated by the critical temperature lines *T*_*V*_(*ρ*), *T*_*Q*_(*ρ*), and *T*_*M*_(*ρ*). Besides the high-*T* disorder state (monopole fluid phase), we describe the other four regions one by one below:

1. The monopole dimer region: as defined earlier, the *T*_*V*_(*ρ*) line marks the transitions from the monopole fluid state into the well-ordered vacuum state (occupied mostly with the V_1_ vertices) plus monopole dimers, assigned as region (b) for relatively low *ρ* (*ρ* < *ρ*_*C*_). A typical lattice configuration in this region is shown in Fig. 9(b) where the monopole dimers (circled for guide of eyes) are randomly distributed in the ordered vacuum matrix.
2. The staggered charge-order (SCO) region: the *T*_*Q*_(*ρ*) line marks the onset of the SCO phase, assigned as region (c) for relatively high *ρ* (*ρ* > *ρ*_*C*_). A typical lattice configuration is presented in Fig. 9(c) where monopole crystals coexist with vacuum phase in the lattice. However, the monopoles on the surfaces of these finite-sized crystals may be movable with time. An additional characteristic of this region is that the vacuum phase is disordered, filled with both the V_1_ and V_2_ vertices. This implies that the magnetic structure in this region is disordered, as seen most clearly from the spin alignments along the two diagonal directions.
3. The low-*T* monopole crystal region: the *T*_*M*_(*ρ*) line is the critical line for the long-range magnetic ordering and this line is always below the *T*_*Q*_(*ρ*) line and the *T*_*V*_(*ρ*) line. The region below this *T*_*M*_(*ρ*) line is assigned as region (e) and a typical lattice configuration is given in Fig. 9(e), where the monopole crystals coexist with the vacuum phase too. However, in this case, the monopole crystals show well-defined shape such as rectangle or square. In the other words, the monopoles on the surfaces of the crystals are no longer movable and they always align along one of the two diagonal directions. At the same time, the vacuum phase is also well ordered and occupied by the V_1_ vertices alone.
4. The transition region: the three critical lines *T*_*V*_(*ρ*), *T*_*Q*_(*ρ*), and *T*_*M*_(*ρ*) bound a finite region assigned as region (d) which has very narrow *T*-range. The details of monopole state and vacuum state in this region depend on the location of the lattice in the phase diagram. For example, for relatively high *ρ*, this region shows well-ordered vacuum phase which is similar to that in region (e) but different from that in region (c), i.e. the vacuum phase is mostly occupied by the V_1_ vertices. On the other hand, the monopoles on the surfaces of the crystals are also movable, similar to that in region (c) but different from that in region (e). What should be mentioned is that a full understanding of every particular in this region in terms of the spin and charge ordering behaviors needs more detailed investigations, which is beyond the scope of this work.

### Remarks and experimental relevance

So far we have presented our comprehensive simulations on the phase diagram of square spin ice lattice over the whole *ρ-T* space and a series of magnetic and magnetic-charge phase transitions have been discussed. Nevertheless, our simulations are based on the conserved monopole density algorithm, which may not be fully compatible with practical spin ice systems including artificial spin ices. Also, quite a few of crucial issues beyond earlier works and the present simulations may be discussed too. The experimental relevance of the simulated results is also concerned. We only discuss several issues for pyrochlore spin ices and artificial spin ices which are relevant with the present simulations.

#### Pyrochlore spin ices

For pyrochlore spin ices, earlier works demonstrated that the effective nearest-neighbor interaction is highly sensitive to the lattice constant^14^,^20^,^39^. For example, when the lattice constant has a reduction of ~1.68%, from Dy_2_Ti_2_O_7_ to Dy_2_Ge_2_O_7_, the effective nearest-neighbor interaction decreases from 1.20 K to 0.67 K^14^. This implies that a uniaxial pressure along the^19^ axis would split the effective nearest-neighbor interaction into two sets, corresponding to the *J*_1_ and *J*_2_ in our model. The six spin ice states are also split into two sets according to their energy non-degeneracy. Experimentally, a 1% lattice shrinkage of both Ho_2_Ti_2_O_7_^20^ and Dy_2_Ti_2_O_7_^39^ was successfully realized by applying physical pressure, giving rise to a considerable energy gap between the two sets of ice states (e.g. Δ/*D* ~ 0.12 for Ho_2_Ti_2_O_7_). Such anisotropic spin ice is closely related to the square spin ice model.

In addition, fully magnetized spin ice phase was reported in such anisotropic spin ice systems^40^. The phase transition from monopole liquid to this magnetized phase is of the first-order, corresponding to the transition from monopole fluid phase to monopole dimer phase in the present monopole conserved model. As shown in our simulated results, the energy splitting of the six ice states can enhance the binding energy of monopole dimers. Considering the fact that the monopole density can be increased by lowering the chemical potential down to zero^41^,^42^, one can expect the monopole-antimonopole dimer pairs predicted in our simulations in realistic anisotropic pyrochlore spin ices.

#### Artificial spin ice

For artificial spin ice^6^,^43^,^44^, the anisotropic barrier of the magnetic islands prevents the spins from free flipping. As a result, the spin configurations are determined by not only the ground energy but also the relaxation process^45^. There is a hopeful route to probe the monopole correlated phenomena experimentally. We may initial a high-Δ system using a magnetic field along the^31^,^45^ axis to obtain a lattice fully filled with the V_2_ vertices by means of micro-fabrication. After the field is removed, the system would relax to the V_1_-ordered ground state. The key to our first proposed experiment is to create a proper high-Δ system. According to equation (7) in the manuscript, the Dirac string energy is proportional to the energy gapΔ, while the monopole excitation energy *c* is proportional to *J*_2_. If the system has a small *J*_2_ but largeΔ, the system intends to relax by creating more monopoles and short Dirac strings in the dynamic process from V2 state to V1 state. This relaxation process allows an access to a spin ice lattice with any pre-assigned monopole density. The monopoles dynamics can be modulated by the Coulomb’s law.

## Conclusion

In conclusion, we have systematically simulated the monopole conserved square spin ice model with conserved monopole density using the Monte Carlo algorithm. In the dilute monopole case (*ρ* → 0), the monopoles dynamics is dominantly controlled by the Coulomb potential. It is revealed that there exists a monopole dimer phase between the critical temperature points *T*_*V*_ and *T*_*M*_, in which the neutral monopole dimer pairs move like the molecular Brownian motion. The staggered charge order phase and monopole crystal phase as a function of monopole density have been discussed in details, and the relevant phase transitions have been carefully characterized using the finite size scaling. The phase diagram over the whole temperature-monopole density space has been constructed by extensive simulations on the magnetic and charge ordering sequences. The present work provides a comprehensive understanding of the emergent phase transitions and monopole dynamics in artificial square spin ices.

## Additional Information

**How to cite this article**: Xie, Y.-L. *et al.* Magnetic-charge ordering and phase transitions in monopole-conserved square spin ice. *Sci. Rep.* **5**, 15875; doi: 10.1038/srep15875 (2015).

## Supplementary Material

Supplementary Information
