# Supplementary material for: Magnetic-charge ordering and phase transitions in monopole-conserved square spin ice
Source: Sci Rep. 2015 Oct 29;5:15875. doi: 10.1038/srep15875 (PMC4625371; doi:10.1038/srep15875)
Supplement: Supplementary Information [file srep15875-s1.pdf]

Supplementary materials to ***Scientific Reports*** **(Ref. SREP-15-25014)**.

Magnetic-charge ordering and phase transitions in monopole-conserved square spin ice

Y. –L. Xie[1], Z. –Z. Du[1], Z. –B. Yan[1], and J. –M. Liu [1,2,*]

[1]*Laboratory of Solid State Microstructures and Innovative Center of Advanced Microstructures, Nanjing University, Nanjing 210093, China*

[2]*Institute for Advanced Materials and Laboratory of Quantum Engineering and Materials, South China Normal University, Guangzhou 510006, China*

[*] Correspondence and requests for materials should be addressed to J. -M. Liu (E-mail: liujm@nju.edu.cn)

1. Simulation details

We employ the Monte Carlo method to track the magnetic states and associated phase transitions in such dipolar square spin ice model with different monopole density ρ. In our simulations, the long-range dipolar interaction is treated using the Ewald summation scheme, avoiding the errors induced by the conventional finite truncation method. In order to track the monopole-ordered phases, we employ the conserved monopole algorithm (CMA)[1], which applies to a statistical ensemble with a fixed monopole density over the whole T-range.

According to the CMA, any spin flip creating or destroying monopole is strictly forbidden. Considering a neighboring V_i-V_j vertex-pair, which is denoted as the $V_{i,j}$-pair, any single spin flip will carry a monopole from one vertex to the other. Therefore, relationship $E_{1,3}=E_{1,4}<E_{2,3}=E_{2,4}$ is satisfied, where $E_{i,j}$ is the energy of the vertex-pair V_i-V_j. It is clear that the spin flip from the $V_{2,3}$-pair to the $V_{1,3}$-pair or from the $V_{2,4}$-pair to the $V_{1,3}$-pair has higher probability than that for the flip from the $V_{1,3}$-pair to the $V_{2,3}$-pair or from the $V_{1,3}$-pair to the $V_{2,4}$-pair, because we have $E_{2,3}-E_{1,3}=E_{2,4}-E_{1,4}=E_2-E_1=\Delta>0$. However, the single spin flip scheme alone is very inefficient to reach the ground state and one may include other schemes which can accelerate the ground state searching in addition to the single spin flips. Here, the loop spin flip method[2] is used. This method will not move the monopoles but is very efficient in bypassing the energy barriers that separate degenerate states and allows efficient tracking the long-range ordered states without violating the ice rule. Besides, the loop spin flip is a prominent conserved monopole algorithm because it strictly sticks to the flip rules of CMA.

The simulation starts from an initial state with fixed monopole defects (i.e. fixed and equivalent numbers of V_3 vertex and V_4 vertex). Such initial state can be obtained by the following procedure. We first fill up the lattice with the V_1 vertices, and then run the spin flips which can create new monopoles until a desired monopole density (ρ) in the lattice is reached. Subsequently, we allow sufficient loop spin flip events to reach a random state as the initial state. In the following Monte Carlo steps, we employ the parallel temperature Monte Carlo scheme [3] which is very efficient for complicated spin systems and frequently used in parallel algorithm. Each Monte Carlo step in our simulation contains $N/2$ single spin flips and $N/2$ loop spin flips, where N is the lattice size (L^2). At each temperature point, we complete 2×10^5 MC steps for relaxation process and perform 1×10^5 sampling averages in the next 5×10^5 MC steps.

2. Physical properties of the system in the ρ =0.5 case

Here we present some of our simulation results at the case ρ =0.5. Figure S1 shows the specific heat and the order parameters of the system. There are three order parameters in our system,

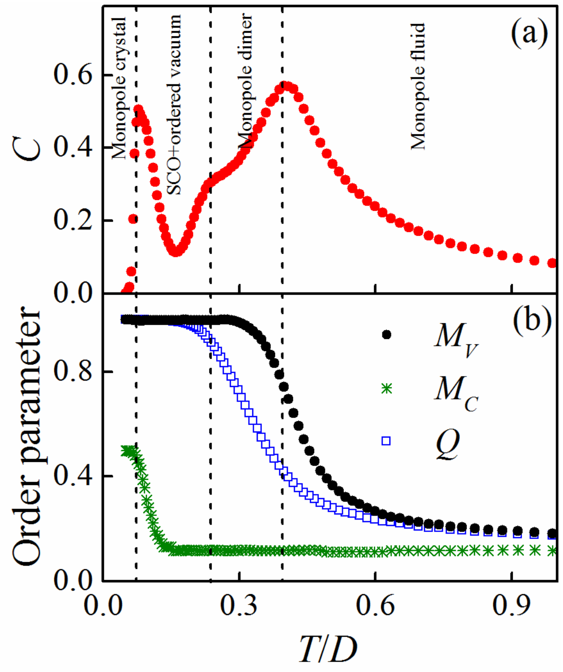

Figure S1. (color online) (a) Specific heat C and (b) order parameters (M_V, M_C, Q) as a function of T for ρ =0. 5 at a lattice size L=32.

and they are the vacuum order parameter (M_V), charge crystal order parameter (M_C) and the staggered charge order parameter (Q). Three peaks in the specific heat curve indicate that the system goes through three phase transitions in an annealing process. At the temperature range T/D>0.4, the system is in the monopole fluid state. As the temperature decreases, M_V quickly rises to 1, indicating that the system is in the vacuum ordered state. Furthermore, a phase transition occurs at about 0.23D leading the system to a staggered charge ordered state. When the temperature decreases blow 0.1D, the charge crystal order parameter rapidly rises to maximum, corresponding to a phase transition to the monopole crystal phase.

3. Supplementary materials References:

[1] Borzi, R. A., Slobinsky, D. & Grigera, S. A. Charge Ordering in a Pure Spin Model: Dipolar Spin Ice. *Phys. Rev. Lett.* **111**, 147204 (2013).

[2] Melko, R. G. & Gingras, M. J. P. Monte Carlo studies of the dipolar spin ice model. *J. Phys.: Condens. Matter* **16**, R1277-R1319 (2004).

[3] Hukushima, K. & Nemoto, K. Exchange Monte Carlo method and application to spin glass simulations. *J. Phys. Soc. Jpn.* **65**, 1604-1608 (1996).
